# Seasonal dynamics of intestinal microbiota in juvenile Chinese mitten crab (*Eriocheir sinensis*) in the Yangtze Estuary

**DOI:** 10.3389/fcimb.2024.1436547

**Published:** 2024-07-04

**Authors:** Ze Qin, Sikai Wang, Yeling Wu, Jinhui Sun, Feng Zhao

**Affiliations:** ^1^ College of Fisheries, Tianjin Agricultural University, Tianjin, China; ^2^ East China Sea Fisherises Research Institute, Chinese Academy of Fishery Sciences; Key Laboratory of East China Sea Fishery Resources Exploitation, Ministry of Agriculture, Shanghai, China; ^3^ Shanghai Yangtze River Estuary Fishery Resources Enhancement and Ecological Restoration Engineering and Technology Research, Shanghai, China

**Keywords:** juvenile Chinese mitten crab, intestinal microbiota, Yangtze River estuary, 16S rRNA gene, intestinal microbiota biomarker species

## Abstract

**Introduction:**

In this study, the seasonal differences in the intestinal microbiota of Chinese mitten crab (*Eriocheir sinensis*) larvae were investigated at different sites in the intertidal zone of the Yangtze River Estuary.

**Methods:**

16S rRNA high-throughput sequencing technology was used to compare and analyze the microbial community structure in the intestines of juvenile crab from different seasons.

**Results:**

The results showed that the main microbial phyla in all seasons and sites were Proteobacteria, Bacteroidetes, Firmicutes, and Actinobacteria, which accounted for 97.1% of the total microbiota. Composition analysis revealed that the relative abundance of Proteobacteria decreased from summer to winter at each station, whereas Bacteroidetes showed the opposite trend. Alpha diversity analysis showed that species richness increased from summer to winter at the upstream site (*P* < 0.05), but decreased at the downstream site (*P* < 0.05), with no significant differences observed in other comparisons. Biomarker species analysis showed that juvenile crab exhibited a more specialized microbial community in summer compared with autumn and winter. Co-occurrence network analysis revealed that microbial interaction network complexity was lower in autumn compared with summer and autumn. Functional prediction analysis showed that the microbial community only exhibited seasonal differences in amino acid biosynthesis, cofactor, prosthetic group, electron carrier, and vitamin biosynthesis, aromatic compound degradation, nucleotide and nucleoside degradation, and tricarboxylic acid cycle pathways.

**Discussion:**

The results indicated that the microbiota did not significantly differ among sites, and seasonal variation was a main factor influencing the differences in intestinal microbiota of Chinese mitten juvenile crab. Moreover, the microbial community was more complex in summer compared with autumn and winter.

## 1 Introduction

The intestinal microbiota of organisms is one of the most influential symbiotic communities, significantly contributing to host health by regulating host metabolism, nutrient absorption, energy utilization and storage, mucosal modulation, immune function, disease prevention, and host development and reproduction (Guo et al., 2021; Xia et al., 2021; Wong et al., 2023). Although population divergence and ecological adaptive potential are typically influenced at the genomic level, reports have suggested that the intestinal microbiome may also drive host population divergence. Changes in the composition of the intestinal microbiota during host evolution may lead to functional alterations, thereby modulating host nutrition, environmental adaptability, and phenotypic plasticity (Lindsay et al., 2020). Currently, there is growing interest in exploring the relationship between the microbiome and host adaptability, making microbial research a focal point in ecology. Because the intestinal microbiome appears to shape the host’s adaptive potential through its compositional changes, a comprehensive understanding of intestinal microbial communities and their underlying mechanisms is crucial for understanding host adaptability (Colston and Jackson, 2016).

Human activities may lead to habitat reduction for wildlife, with many populations confined to the margins of their original natural ranges (Li Q. et al., 2023). Animals are vulnerable to changes in food resources and environmental temperature. Wildlife face temporal variations in food availability and often adjust their diets accordingly, yet the extent to which this adjustment affects the intestinal microbiota is poorly understood (Ren et al., 2017). A previous study suggested that changes in the intestinal microbiota of wildlife may represent an adaptive mechanism for obtaining adequate nutrition during seasonal fluctuations in response to changes in food availability (Rothman et al., 2008). Additionally, the intestinal microbiota of animals indirectly responds to environmental temperature and is directly influenced by the host’s physiological responses to seasonal changes in food sources (Greene et al., 2019). Seasonal variations in host diet may also lead to changes in metabolic pathways, consequently resulting in functional and compositional differences of the intestinal microbiota (Li et al., 2022).

The Chinese mitten crab, Eriocheir sinensis (Grapsidae: Varuninae), is an economically important species in China. The Yangtze River Estuary is the breeding ground for this species. Every December, adult crabs migrate to the estuarine waters of the Yangtze River Estuary to mate and lay fertilized eggs, which develop into larval stages known as zoeae. After five molting stages, they develop into megalopae, and after five more molting stages, they become juveniles (Geng, 2018). During metamorphosis, which occurs in late May to early June, they migrate to the nearshore waters of the Yangtze River Estuary and use these areas as their habitat (Zhao et al., 2020). In our research investigation, we found that juvenile Chinese mitten crabs can still be found in intertidal habitats until January of the following year, which indicates that the Yangtze River Estuary intertidal habitat provides crucial habitat for the early development of Chinese mitten crabs.

Intestinal microbiota and animal hosts have evolved into inseparable “life communities,” with the microbiota influencing the host’s physiology, reproduction, metabolism, and phenotypic traits (Gao et al., 2023). The predominant phyla of crustacean intestinal microbiota include Proteobacteria, Firmicutes, Bacteroidetes, and Actinobacteria (Mente et al., 2016; Holt et al., 2021; Foysal, 2023). Previous studies revealed that the intestinal microbiota of Chinese mitten crabs may be influenced by both the host’s own phylogenetic system and external changes. During the migration of early stage larvae from seawater to freshwater, the relative abundance of main phyla all exhibited significant and temporal patterns. Specifically, Proteobacteria showed decreased trends over time during seawater-freshwater migration, while Firmicutes and Bacteroides showed an increasing pattern (Shao et al., 2022). In wild Chinese mitten crabs from the Yangtze River Estuary, the proportion of Firmicutes in the intestinal microbiota increases while the proportion of Proteobacteria decreases after parental crabs lay eggs (Jing et al., 2021). In artificial aquaculture environments, the intestinal microbiota of juvenile Chinese mitten crabs varies at different times within a day, and most of the changes are in the proportions of Proteobacteria and Firmicutes (Yu et al., 2021).

This study explored the seasonal differences in the intestinal microbiota of Chinese mitten crab juveniles in the Yangtze River Estuary. Continuously monitoring juvenile crabs and analyzing their intestinal microbiota in different seasons and locations of the Yangtze River Estuary provided insight into the mechanisms by which these crabs maintain a stable composition and structure of their intestinal microbiota in response to seasonal variations. These results can be used to help infer the adaptive relationship between the intestinal microbiota and hosts in response to environmental changes.

## 2 Materials and methods

### 2.1 Study area and sampling methods

**Figure 1:**
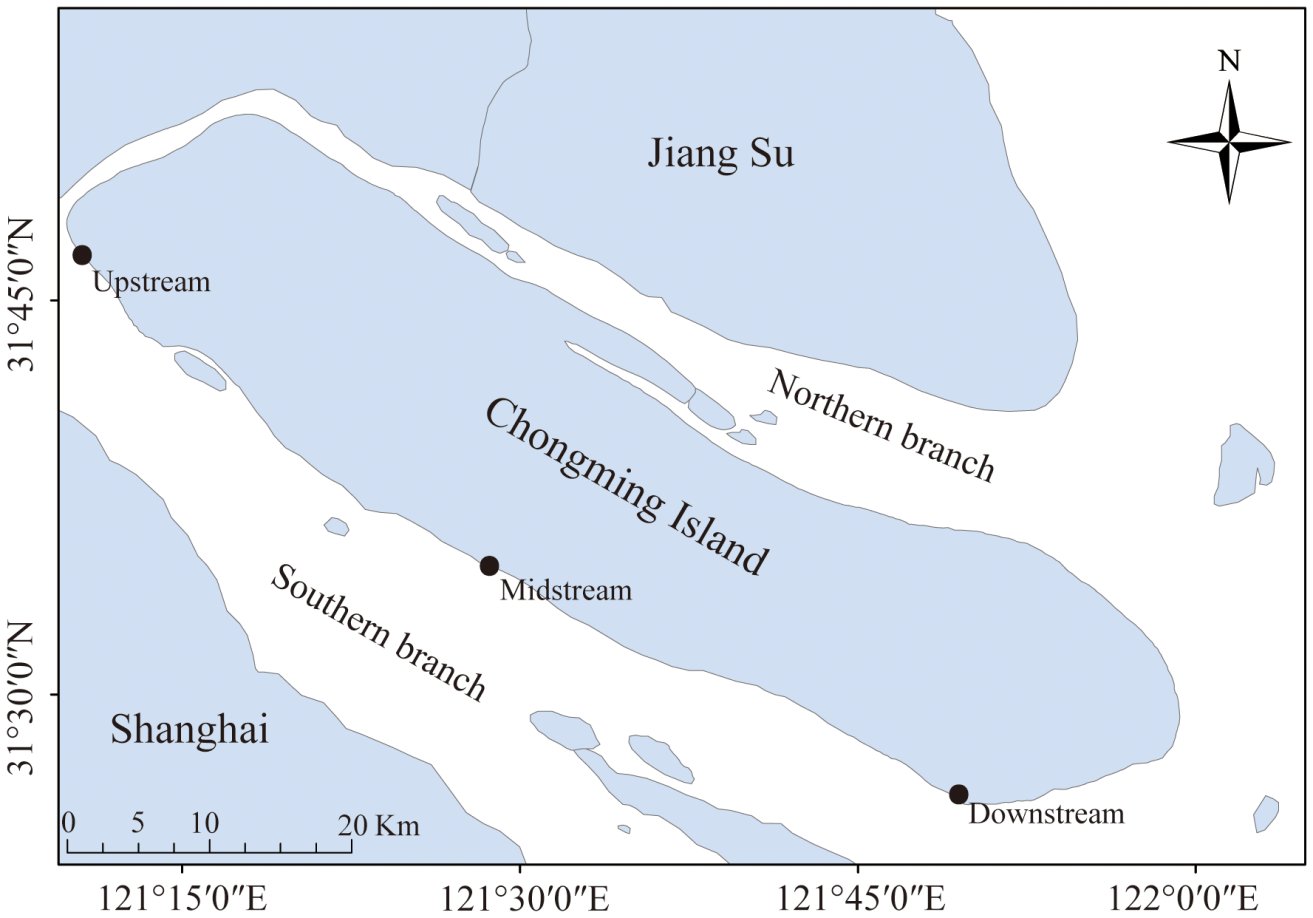
Sample sites, southern branch of Yangtze Estuary, China. Sampling of juvenile Eriocheir sinensis occurred in the upstream, midstream, and downstream reaches of the estuary.

The study was carried out in the Chongming island in the Yangtze River Estuary, the largest estuary in China (**Figure 1**). It lies within a typical semitropical monsoon region, where the climate is mild and wet, and it experiences four distinct seasons. Tides are semidiurnal and irregular, with the amplitude greatest at the river mouth, decreasing landward and seaward from there, and averaging 2.4–4.6 m within the estuarine system (Wang et al., 2021). Three sampling sites in the southern branch of Yangtze River Estuary were designated: an upstream site, a midstream site, and a downstream site. Crabs were collected during spring tides each month in the summer (June to August), autumn (September to October), and winter (November to December) of 2022. In the intertidal zone of the Yangtze River Estuary, habitats such as rocks, grasses, reeds, and mudflats provide temporary residences for juvenile Chinese mitten crabs. We captured these juveniles by hand in their habitats and stored them in 50-milliliter test tubes. A total of 270 juvenile crabs were collected, with an average carapace length of 5.1 ± 1.7 mm (**Table 1**). The field-collected juvenile crabs were euthanized by freezing. The carapace length of the juvenile crabs was measured using calipers. They were then placed on a petri dish lined with ice, and their intestines and stomachs were dissected using tweezers and stored in test tubes for freezing. For analysis, the stomach and intestine were dissected from every 10 juvenile crabs to create one sample, which resulted in a total of 27 samples for intestinal microbiome analysis.

**Table 1:** Carapace length of juvenile Chinese mitten crabs in different months and different sites.

Month	Upstream		Midstream		Downstream	
	range	mean ± S.E.	range	mean ± S.E.	range	mean ± S.E.
Jun.	2.4–3.5	3.0 ± 0.3	3.0–3.7	3.5 ± 0.2	2.8–4.7	3.5 ± 0.6
Jul.	3.0–4.9	3.9 ± 0.7	2.5–4.4	3.3 ± 0.5	2.81–5.03	4.0 ± 0.7
Aug.	3.5–4.0	3.8 ± 0.2	4.4–6.7	5.6 ± 0.8	4.5–5.6	5.2 ± 0.3
Sep.	4.0–5.7	4.9 ± 0.7	4.2–6.2	5.1 ± 0.6	4.4–5.3	5.1 ± 0.4
Oct.	4.4–5.8	5.2 ± 0.5	8.4–13.2	9.2 ± 1.4	5.8–6.9	6.2 ± 0.
Nov.	5.7–6.7	6.2 ± 0.3	6.3–9.1	7.5 ± 1.1	7.0–9.5	8.0 ± 0.8
Dec.	5.4–7.0	6.1 ± 0.6	5.7–7.3	6.3 ± 0.5	4.9–6.0	5.5 ± 0.4

### 2.2 DNA extraction

Total genomic DNA samples were extracted using the Omega Soil DNA Kit (M5635–02) (Omega Bio-Tek, Norcross, GA, USA) and stored at −20°C until further analysis. The extracted DNA quantity and quality were measured using a NanoDrop NC2000 spectrophotometer (Thermo Fisher Scientific, Waltham, MA, USA) and agarose gel electrophoresis, respectively.

### 2.3 Amplification and sequencing of 16S rRNA and 18S rRNA gene amplicons

The bacterial 16S rRNA gene V3–V4 region was amplified by PCR using the universal primers 338F (5′-ACTCCTACGGGAGGCAGCA-3′) and 806R (5′-GGACTACHVGGGTWTCTAAT-3′), which resulted in an approximately 480-bp fragment. Sample-specific 7-bp barcodes were incorporated into the primers for multiplex sequencing. PCRs consisted of 5 μl of 5× buffer, 0.25 μl of Fast pfu DNA polymerase (5 U/μl), 2 μl of 2.5 mM dNTPs, 1 μl of each forward and reverse primer (10 μM), 1 μl of DNA template, and 14.75 μl of ddH2O. The thermal cycling program included an initial denaturation step at 98°C for 5 min, followed by 25 cycles of denaturation at 98°C for 30 s, annealing at 53°C for 30 s, extension at 72°C for 45 s, and a final extension at 72°C for 5 min. PCR amplicons were purified using Vazyme VAHTSTM DNA Clean Beads (Vazyme, Nanjing, China) and quantified using the Quant-iT PicoGreen dsDNA Assay Kit (Invitrogen, Carlsbad, CA, USA). Following individual quantification, amplicons were pooled in equimolar concentrations, and paired-end sequencing (2× 250 bp) was performed on the Illumina NovaSeq platform using the NovaSeq6000 SP Reagent Kit (500 cycles) by Shanghai Personal Biotechnology Co., Ltd. (Shanghai, China).

### 2.4 Sequence analysis

Bioinformatics analysis of microbial and food composition was conducted using QIIME2 2019.4. Briefly, the raw sequence data were demultiplexed using the demux plugin, followed by primer trimming using the cutadapt plugin. Subsequently, sequences were quality filtered, denoised, and merged, and chimeras removed using the DADA2 plugin. Non-singleton amplicon sequence variants (ASVs) were aligned with mafft (Katoh et al., 2002) and used for phylogenetic tree construction in fasttree2 (Price et al., 2009). The classify-sklearn naïve Bayes classifier in the feature-classifier plugin (Bokulich et al., 2018) was used to classify ASVs according to the Greengenes Release 13.8 database.

### 2.5 Bioinformatics and statistical analysis

Sequence data analysis was primarily performed using QIIME2 and R 3.2.0. Alpha diversity indices at the ASV level, such as Chao1 richness, Observed_species, Shannon diversity index, and Simpson index, were calculated using the ASV table in QIIME2 and visualized as boxplots. Taxonomic composition and abundance were visualized using MEGAN and GraPhlAn.LEfSe (Linear discriminant analysis effect size) was performed to detect differentially abundant taxa across groups using the default parameters (Segata et al., 2011). Because of the complexity of analyzing the many ASVs detected in this study, statistical analysis was performed from the domain to the genus level. The LDA score was set to ≥ 2 for confirmation by LEfSe. Symbiotic Network Analysis was conducted using the R 4.3.1 packages devtools, spiecEasi, and igraph to calculate the strength and direction of relationships between different species. The data was imported into Gephi software to generate the network graph.Microbial functions were predicted by PICRUSt2 (Phylogenetic investigation of communities by reconstruction of unobserved states) (Gavin, et al., 2019) upon MetaCyc (https://metacyc.org/) and KEGG (https://www.kegg.jp/) databases.

## 3 Results

### 3.1 Sequencing results

High-throughput sequencing of the juvenile Chinese mitten crab stomach contents targeting the 16S rDNA gene yielded an average of 92,431 raw sequences. After quality filtering, an average of 90,578 high-quality sequences were obtained. These sequences were then clustered into ASVs, which resulted in a total of 12,829 ASVs (**Table 2**). The representative sequences of the ASVs were compared to the NCBI database to annotate the taxonomic information for each ASV. Excluding those that could not be assigned to any known taxonomic unit, the classified ASVs belonged to 43 phyla, 421 genera, to 638 species.

**Table 2:** Raw reads, high-quality reads, and ASVs in different samples.

Station	Season	Sample ID	Average raw reads	Average high quality reads	Average ASV
Upstream	Summer	USum	89263	87682	404
	Autumn	UAut	93869	90971	486
	Winter	UWin	107226	106560	767
Midstream	Summer	MSum	99276	95769	441
	Autumn	MAut	93777	93012	627
	Winter	MWin	94217	92434	345
Downstream	Summer	DSum	92902	89302	751
	Autumn	DAut	78226	74599	669
	Winter	DWin	93781	92304	394

### 3.2 Composition of core microbial taxa

**Figure 2:**
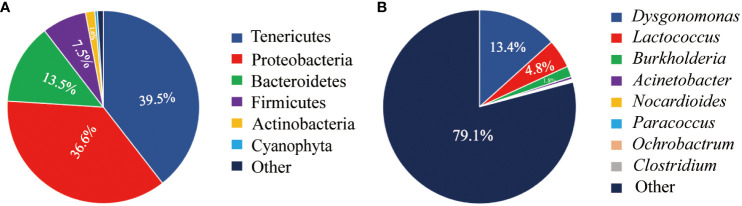
Abundance of intestinal microbiota at the phylum level **(A)** and genus level **(B)** in juvenile Chinese mitten crab of the Yangtze River Estuary.

A total of 43 phyla of intestinal microbiota were identified in juvenile Chinese mitten crab (**Figure 2A**). Among these, five dominant phyla with abundances exceeding 1% were identified. Tenericutes was the most abundant phylum, accounting for 39.5% of the total microbiota, followed by Proteobacteria at 36.6%. Additionally, Bacteroidetes accounted for 13.5%, Firmicutes accounted for 7.5%, and Actinobacteria accounted for 1.6%.

This study identified a total of 421 classifiable genera of intestinal microbiota in Chinese mitten crabs (**Figure 2B**). The top genera, ranked by abundance, were *Dysgonomonas*, *Lactococcus*, *Burkholderia*, *Acinetobacter*, *Nocardioides*, *Paracoccus*, *Ochrobactrum*, and *Clostridium*. Among them, *Dysgonomonas*, *Lactococcus*, and *Burkholderia* were the dominant genera, each accounting for over 1% of the total abundance, with proportions of 13.4%, 4.85%, and 1.67%, respectively.

### 3.3 Spatiotemporal differences in intestinal microbiota composition

**Figure 3:**
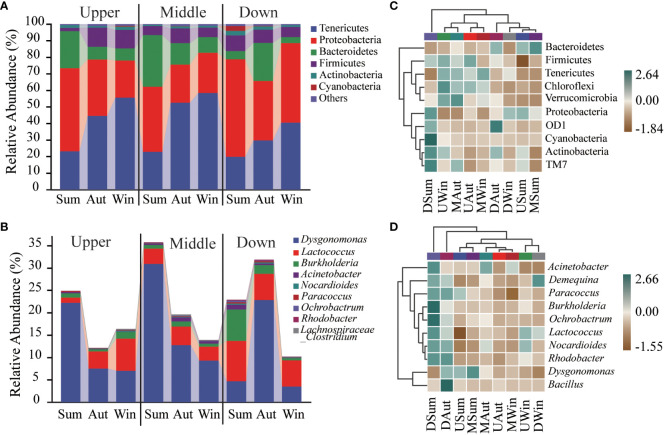
Intestinal microbiota composition of Chinese mitten crab juveniles at the phylum **(A)** and genus **(B)**, levels, and heatmap representation at the phylum **(C)** and genus **(D)** levels.

In the intertidal zone of the Yangtze River Estuary, the relative abundance of intestinal microbiota in Chinese mitten crab juveniles showed significant seasonal variations (**Figure 3**; **Table 3**). The phylum Proteobacteria was most abundant during the summer and decreased during autumn and winter (59.0% to 39.3%). At the upstream site, the relative abundance during winter (22.5%) was significantly lower than that during summer (50.3%, *P* < 0.05). At the midstream and downstream sites, the lowest relative abundance was observed during autumn, with values of 23.0% and 35.8% (*P* < 0.05), respectively. In contrast, the phylum Tenericutes exhibited a gradual increase in relative abundance from summer to winter. The relative abundances at the upstream site were 23.1%, 44.6%, and 55.6% in summer, autumn, and winter, respectively, whereas those at the midstream site were 22.9%, 52.6%, and 58.4%, and those at the downstream sites were 19.8%, 29.8%, and 40.6%. The phylum Bacteroidetes was most abundant during summer at the upstream and midstream sites (22.4% and 31.0%, respectively) and least abundant during winter (7.3% and 9.4%, respectively, *P* < 0.05). Alternatively, at the downstream site, Bacteroidetes was highest during autumn (23.0%, *P* < 0.05) and relatively lower during summer (5.0%) and winter (3.6%).

**Table 3:** Average proportions of different phyla in the intestinal microbiota of Chinese mitten crab juveniles.

Group	Tenericutes	Proteobacteria	Bacteroidetes	Firmicutes
USum	23.1% ± 4.6%^bc^	50.1% ± 7.4%^ab^	22.4% ± 7.1%^ab^	1.9% ± 0.4%^b^
UAut	44.6% ± 3.7%^ab^	34% ± 8.7%^bc^	7.6% ± 8.6%^bc^	11.7% ± 5.9%^a^
UWin	55.6% ± 18.1%^a^	22.5% ± 15.3%^c^	7.3% ± 12%^bc^	11.2% ± 4.7%^a^
MSum	22.9% ± 5.2%^bc^	39.3% ± 6.5%^bc^	31.1% ± 10.4%^a^	5.5% ± 5.1%^ab^
MAut	52.6% ± 23.7%^a^	23% ± 12%^c^	12.9% ± 16.4%^bc^	8.9% ± 4.9%^ab^
MWin	58.4% ± 22.1%^a^	24.3% ± 15.8%^c^	9.4% ± 12.5%^bc^	5.7% ± 2.3%^ab^
DSum	19.8% ± 9.9%^c^	59% ± 15.2%^a^	5% ± 6.1%^c^	9.4% ± 8.5%^a^
DAut	29.8% ± 5.8%^bc^	35.8% ± 11.4%^bc^	23% ± 8.1%^ab^	8% ± 2.2%^ab^
DWin	40.6% ± 6.1%^abc^	48% ± 4.6%^ab^	3.6% ± 0.9%^c^	6.2% ± 2.8%^ab^

Different letters indicate significant differences (*P* < 0.05) in the same taxa between different seasons or sites.

The relative abundance of Proteobacteria during summer was lowest in the midstream site (39.3%) compared with the upstream (50.1%) and downstream (59.0%, *P* < 0.05) sites. However, during winter, Proteobacteria abundance was highest at the downstream site (48%, *P* < 0.05). The relative abundance of Tenericutes at the downstream site (29.8%) was only significantly lower than at the upstream and midstream sites during autumn (*P* < 0.05). Similarly, during summer, the relative abundance of Bacteroidetes was lower at the downstream site (5.0%, *P* < 0.05) than at the upstream and midstream sites.

Taxonomic abundance at the phylum level across different seasons and sites was supported by the corresponding heatmap (**Figure 3C**), which further confirmed seasonal variations in the intestinal microbiota community structure of the juvenile crab. At the upstream site, the abundances of Firmicutes, Tenericutes, Chloroflexi, and Verrucomicrobia decreased from summer to autumn; however, at the midstream site, the abundances of Verrucomicrobia and Firmicutes were higher in autumn compared with summer and winter. At the downstream site, the abundances of Proteobacteria, OD1, Cyanobacteria, and Actinobacteria decreased from summer to winter.

At the genus level, the heatmap of the juvenile crab intestinal microbiota also displayed similar changes (**Figure 3D**). At the upstream site, the abundances of *Paracoccus* and *Dysgonomonas* decreased from summer to winter, whereas those of *Lactococcus* and *Nocardioides* increased. At the midstream site, the abundance of *Dysgonomonas* gradually decreased from summer to winter, whereas the abundances of *Acinetobacter* and *Deinococcus* were higher in autumn than summer and winter. At the downstream site, the abundances of *Burkholderia*, *Ochrobactrum*, *Acinetobacter*, *Paracoccus*, and *Rhodobacter* gradually decreased from summer to winter.

### 3.4 Alpha diversity analysis of intestinal microbiota

**Figure 4:**
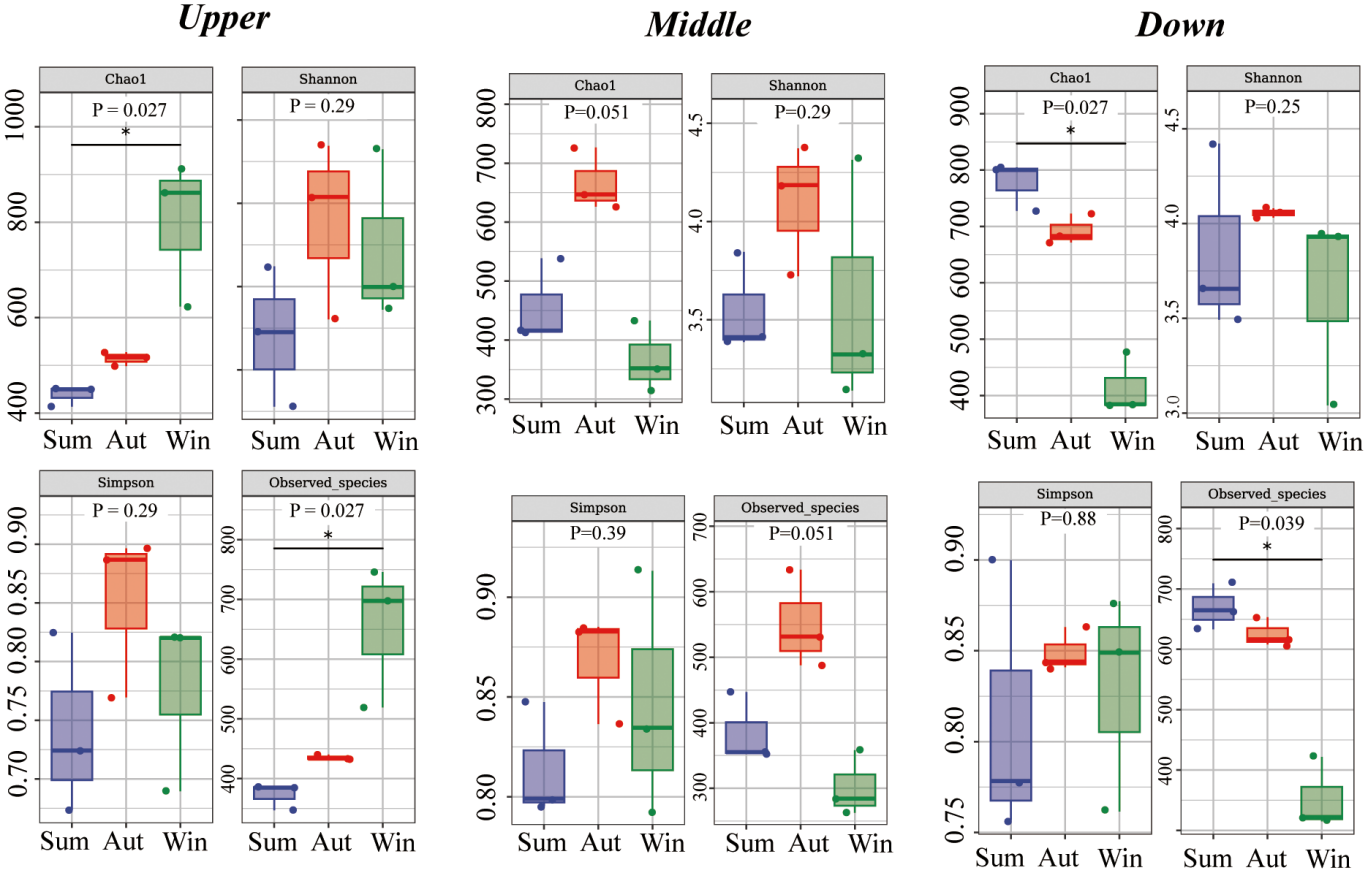
Alpha diversity indices (Chao1, Shannon, Simpson, Observed_species) of intestinal microbiota. Upstream, midstream, and downstream represent comparisons between different seasons at the same site. *P* values are from Kruskal–Wallis tests.

**Figure 5:**
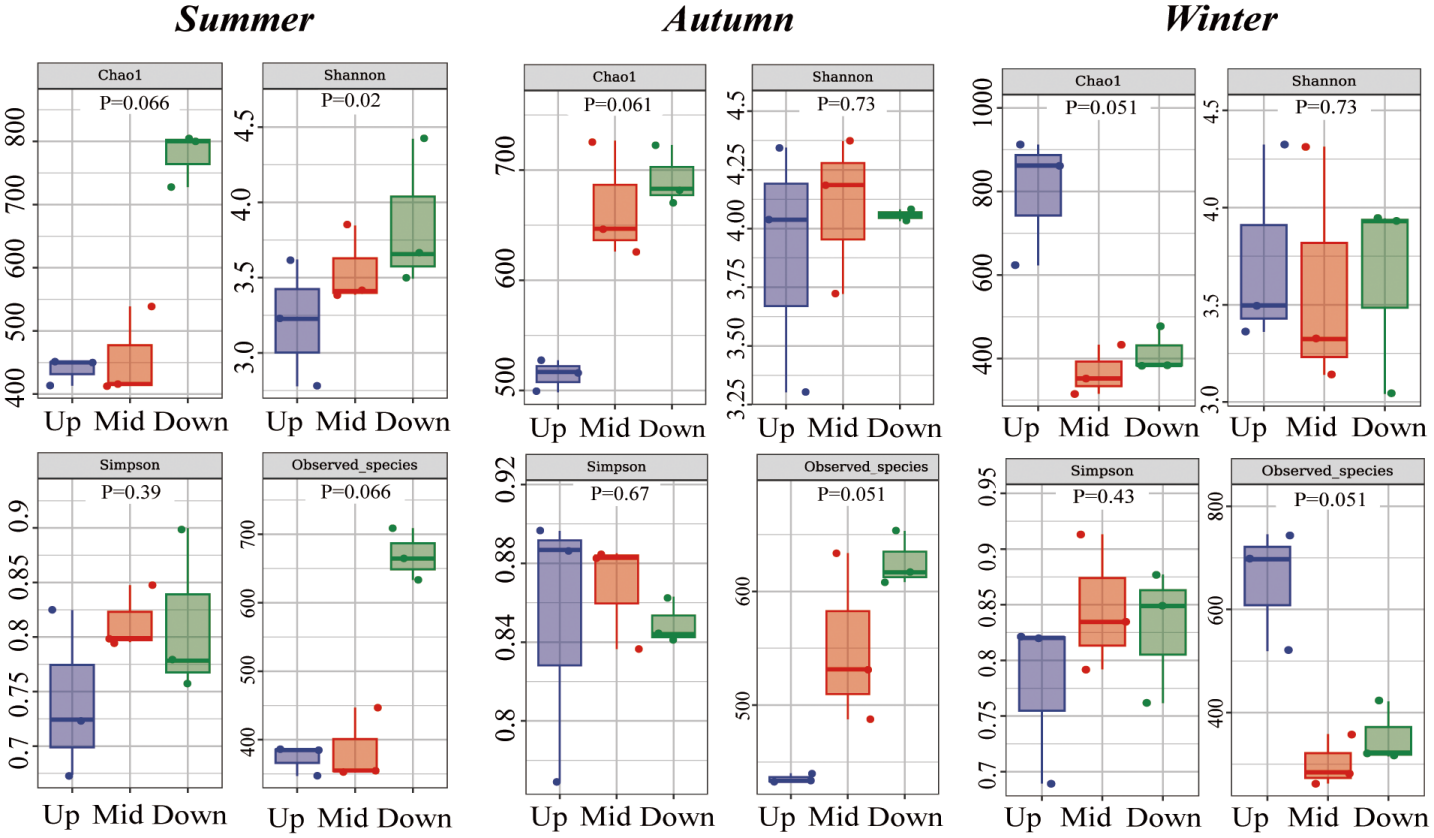
Alpha diversity indices (Chao1, Shannon, Simpson, Observed_species) of intestinal microbiota. Summer, autumn, and winter represent comparisons between different sites within the same season. *P* values are from Kruskal–Wallis tests.

Alpha diversity indices were used to calculate bacterial richness and diversity at the same site during different seasons and at different sites during the same season (**Figure 4**). At the upstream site (Chao1, p=0.027; Faith_pd, Observed_species, *P* = 0.027), species richness increased from summer to winter. There were no significant differences in the indices at the midstream site. At the downstream site (Chao1, p=0.027; Observed_species, *P* = 0.039), species richness decreased from summer to winter. The Shannon index showed no significant differences in microbial diversity among sites and seasons (**Figure 5**).

### 3.5 Analysis of intestinal microbiota biomarker species

**Figure 6:**
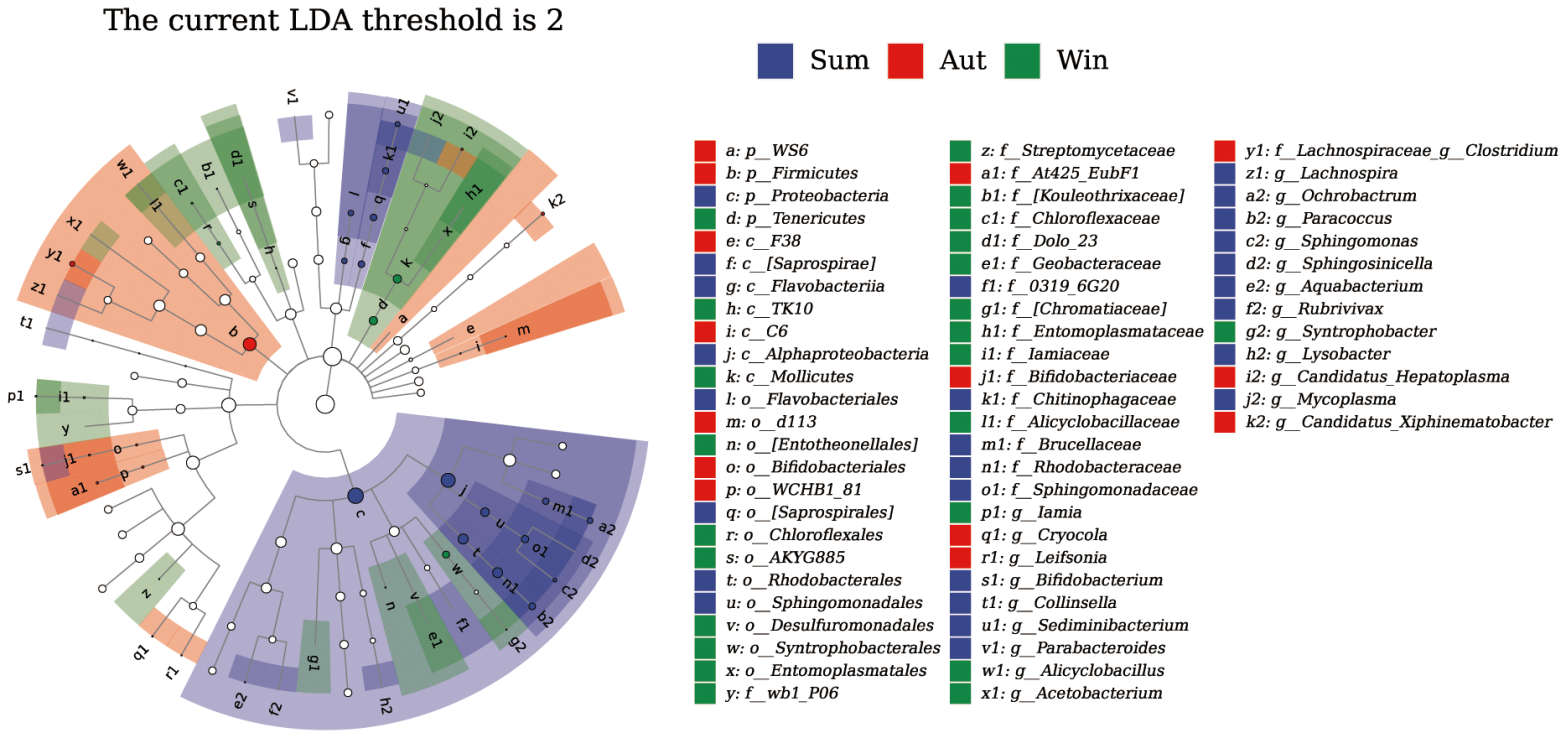
LEfSe analysis of the juvenile Chinese mitten crab intestinal microbiota.

**Figure 6** shows that, during the summer, Proteobacteria were significantly enriched, specifically the order Flavobacteriales, family Sediminibacterium, and genera *Ochrobactrum*, *Paracoccus*, and *Sphingomonas*. In autumn, Firmicutes were enriched, specifically family Lachnospiraceae and genus *Clostridium*, whereas in winter, Tenericutes were enriched, specifically class Mollicutes, order Syntrophobacterales, and genus *Candidatus_Xiphinematobacter*.

### 3.6 Symbiotic network analysis

**Figure 7:**
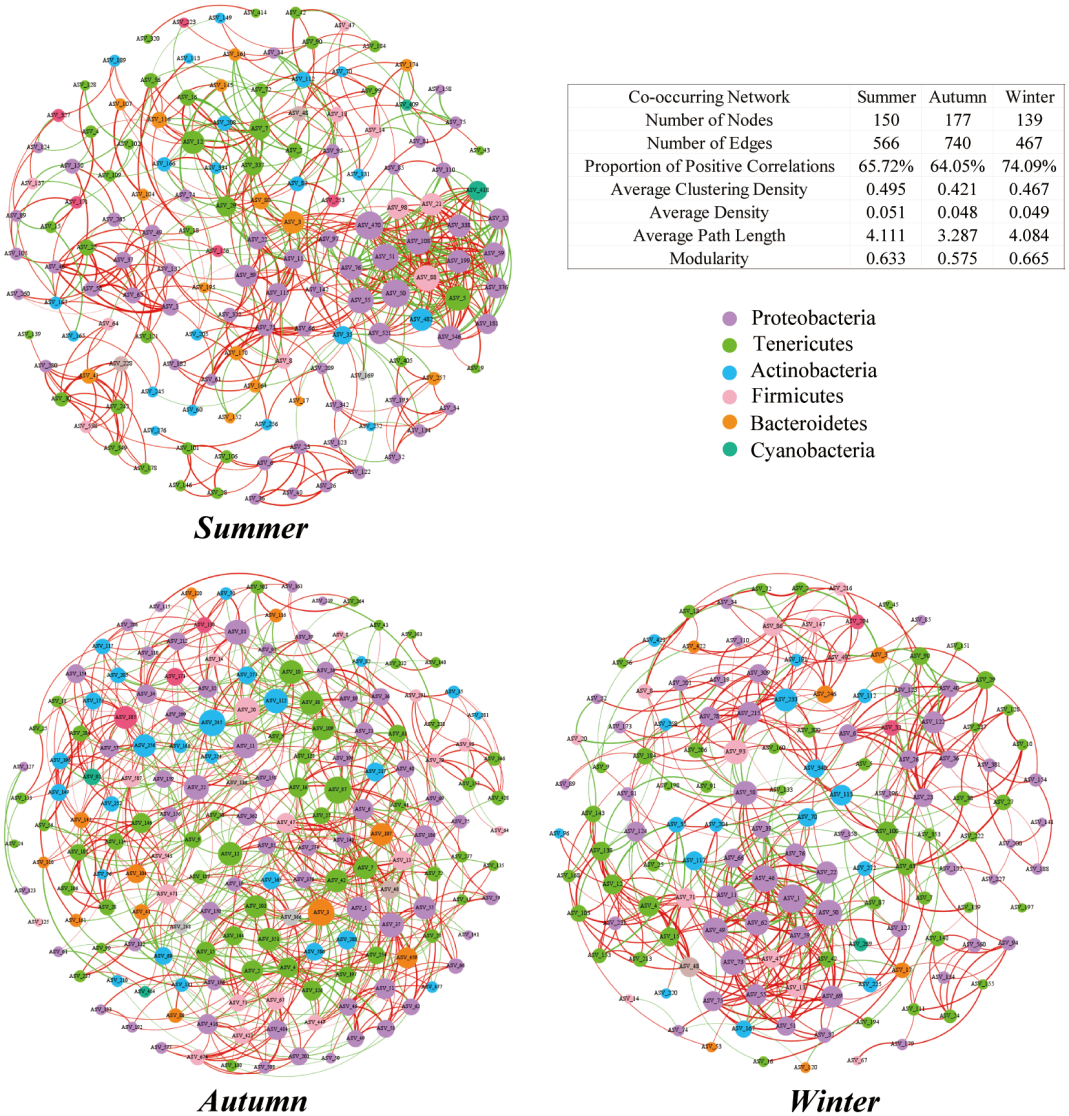
Symbiotic network of juvenile Chinese mitten crab intestinal microbiota at the ASV level. Each node represents a unique ASV in the dataset, and the size of each node is proportional to the number of connections. ASVs are colored according to phylum, with red lines indicating positive correlations and green lines indicating negative correlations.

The intestinal microbiota of the juvenile crab showed the number of nodes and edges was highest in autumn and lowest in winter. Modularity did not significantly differ between summer and winter, but decreased in autumn. The proportion of positive correlations in each network was higher than 60%, which was significantly higher than the proportion of negative correlations, and the highest value (74.61%) occurred in winter (Pearson correlation coefficient r > 0.6, *P* < 0.05). A symbiotic network was established, with Firmicutes and Proteobacteria jointly accounting for 63.3%, 63.9%, and 72.7% of the network in summer, autumn, and winter, respectively; Proteobacteria accounted for 40%, 33.9%, and 33.81%, whereas Firmicutes accounted for 23.3%, 30.5%, and 33.8% (**Figure 7**).

### 3.7 Functional analysis of intestinal microbiota

**Figure 8:**
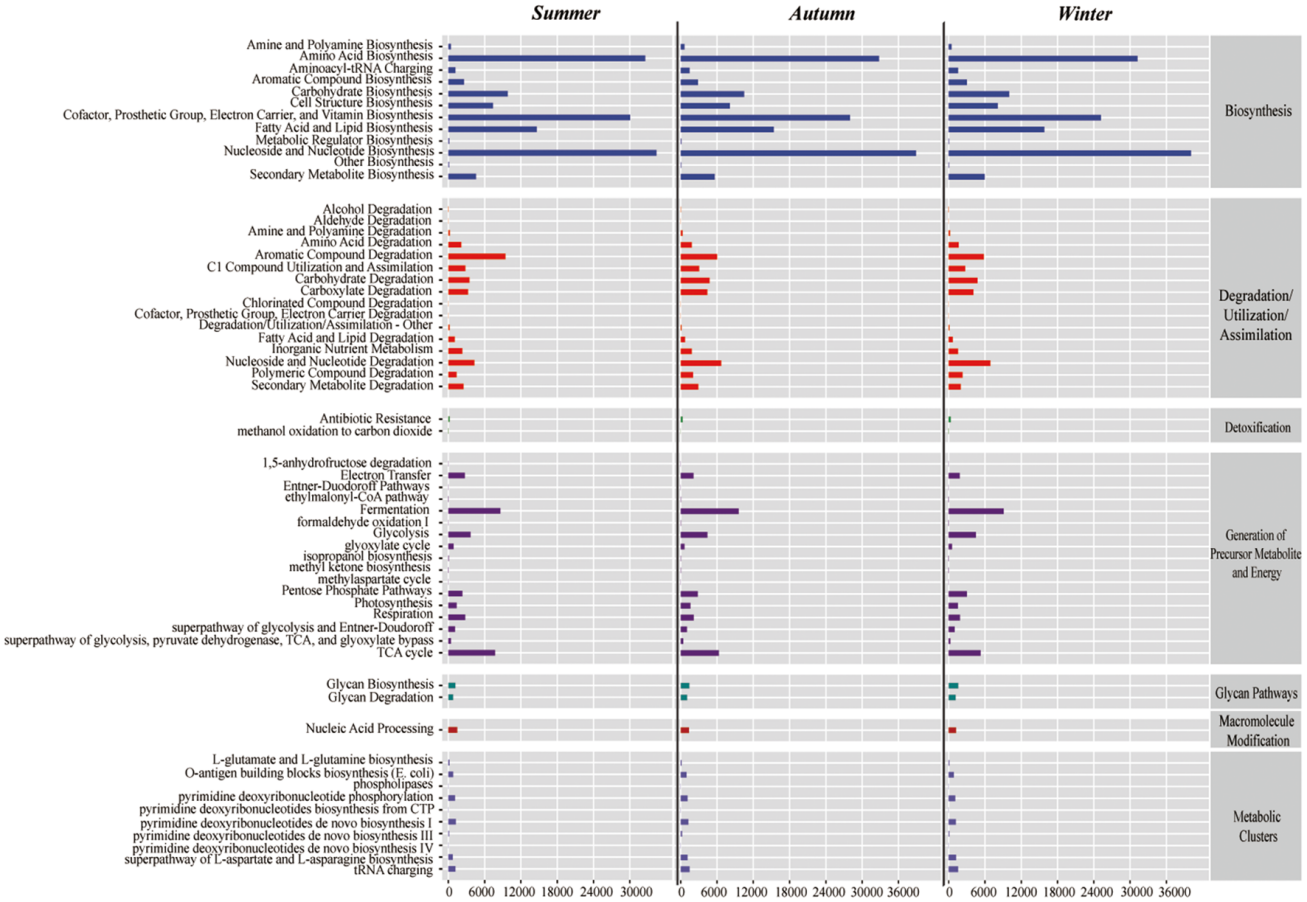
Metabolic pathway analysis of Chinese mitten crab juvenile intestines in summer (top), autumn (middle), and winter (bottom).

In the intestinal microbiota of the juvenile Chinese mitten crab analyzed from the Yangtze River Estuary, the biosynthesis pathway category had the richest pathways, which dominated across seasons. These pathways included nucleoside and nucleotide biosynthesis; amino acid biosynthesis; cofactor,prosthetic, electron carrier, and vitamin biosynthesis. Several other relatively abundant pathways included aromatic compound degradation, nucleoside and nucleotide degradation, and tricarboxylic acid cycle (**Figure 8**).

## 4 Discussion

### 4.1 Intestinal microbiota composition of juvenile Chinese mitten crab

The dominant phyla of intestinal microbiota in juvenile Chinese mitten crab from the intertidal zone of the Yangtze River Estuary were Tenericutes, Proteobacteria, Bacteroidetes, and Firmicutes. Similar compositions of intestinal microbiota were reported in studies conducted in other regions. For example, at crab breeding farms in Chongming Island and Songjiang District in Shanghai of China, and Zhuling City in Jilin Province of China, the predominant phyla of juvenile crab intestinal microbiota were Tenericutes, Bacteroidetes, Firmicutes, and Proteobacteria (Yang et al., 2019; Sun et al., 2020; Zhang et al., 2022).

However, there were differences in Chinese mitten crab intestinal microbiota in some regions. For example, in samples of Chinese mitten crab juveniles collected from four locations (Jintan City in Jiangsu Province, the Teaching and Research Base of Nanjing Agricultural University, Nanjing Gucheng Lake, and overwintering ponds in Liaoning Province), the predominant phyla of the intestinal microbiota were Firmicutes, Proteobacteria, Bacteroidetes, and Tenericutes (Yu et al., 2021; Jiang et al., 2023).

Moreover, research on mud crab (*Scylla paramamosain*) populations indicated that there were differences in the intestinal microbiota composition across different regions. The intestinal microbiota of mud crabs purchased from fishermen in various provinces, such as Zhejiang, Fujian, Guangdong, Guangxi, and Hainan, were primarily composed of the phyla Proteobacteria, Firmicutes, Bacteroidetes, and Actinobacteria (Wei et al., 2019). Comparison of the intestinal microbiota composition of mud crabs among different regions showed that there were differences in the species composition at the phylum level.

The observed intestinal microbiota composition of Chinese mitten crabs was influenced by various factors such as developmental stage and regional differences. In this study, the sampling locations were on Chongming Island; the differences in intestinal microbiota composition among the upper, middle, and lower reaches of the river were not significant and cannot be used to infer regional differences. The phylum-level differences of Chinese mitten crab intestinal microbiota observed in different regions or developmental stages were mainly variations in the abundances of major phyla such as Proteobacteria, Firmicutes, Bacteroidetes, and Tenericutes. However, understanding the influence of developmental stage and regional differences on Chinese mitten crab intestinal microbiota composition requires further validation through subsequent sampling and investigation.

Chongming Island, located at the mouth of the Yangtze River, experiences frequent water exchange, and its natural environment harbors a more diverse microbial community compared with the aquaculture environment. The intestinal microbiota composition of Chinese mitten crabs collected in this study lacked microbial taxa with high relative abundance observed in aquaculture. In this experiment, *Dysgonomonas* exhibited the highest relative abundance at the genus level. Similarly, *Dysgonomonas* was previously observed in the intestinal microbiota of Chinese mitten crab juveniles (Sun et al., 2020). Members of *Dysgonomonas* are gram-negative, non-motile, facultative anaerobic rod-shaped bacteria that are widely distributed in terrestrial environments. They are particularly abundant in insect systems and play important roles in aiding termite digestion of lignocellulose and their immunity and reproduction (Fraune and Bosch, 2010; Su et al., 2016). *Dysgonomonas* is considered a potential probiotic (Wang et al., 2020) and is typically cultured under anaerobic conditions on complex media containing blood, peptone, pancreatin, and yeast, plant, or meat extracts (Bridges and Gage, 2021). Microorganisms of the genus *Dysgonomonas* were also detected in the intestinal microbiota of the Chinese mitten crab which like termites is an arthropod. We speculate that the Chinese mitten crab may consume detritus from plants such as Phragmites and reeds growing in the intertidal zones of Chongming Island, these microorganisms may play a role in the digestion process. Whether *Dysgonomonas* plays an important role in juvenile crabs foraging for food in the wild should be further investigated.

The alpha diversity indices Chao1 and Observed_species showed that there was seasonal variation in the species richness of the intestinal microbiota at the upstream and downstream sites. However, the trends differed between the upstream and downstream sites: the indices increased from summer to winter at the upstream sites, whereas they decreased at the downstream sites. Alpha diversity analysis revealed no differences between the sites during the same season.

### 4.2 Biomarker species differences

LEfSe analysis revealed that, in summer, the intestinal microbiota of the juvenile Chinese mitten crabs was more specialized and primarily included the phylum Bacteroidetes, order Xanthomonadales, and genera *Sediminibacterium*, *Sphingobium*, *Streptomyces*, and *Pseudomonas*. In contrast, in autumn, *Bacillus cereus* became the predominant specialized member of the microbial community. During winter, the specialized microbial community mainly included members of the class Flavobacteriia and order Burkholderiales.

The phyla Firmicutes, Proteobacteria, and Bacteroidetes are the predominant indigenous bacteria that shape the intestinal microbiota composition of Chinese mitten crabs (Zhang et al., 2022). Firmicutes are commonly found in the intestines of marine invertebrates. The Chinese mitten crab transitions from marine to freshwater habitat during its transformation from larval to juvenile stages (Wei et al., 2019), yet Firmicutes did not exhibit high relative abundance in our study. Firmicutes can degrade and metabolize complex carbohydrates, polysaccharides, sugars, and fatty acids to provide energy (Shao et al., 2022). Certain Firmicutes species contribute various enzymes that facilitate digestion and nutrient absorption in Osteichthyes. Bacteroidetes are obligate anaerobes, whereas Proteobacteria are facultative anaerobes. An increase in Proteobacteria can disrupt the oxygen balance in fish intestines, and the intestinal microbiota composition is regulated based on intestinal oxygen levels; this leads to a further increase in Proteobacteria and decrease in Bacteroidetes (Li R. et al., 2023). In this study, from summer to winter, the specialized phyla of the crab intestinal microbiota shifted from Proteobacteria to Bacteroidetes. This was possibly influenced by changes in oxygen balance and reflects potential alterations in the digestive function of Chinese mitten crabs due to changes in intestinal microbial composition.

During the summer, potential probiotic bacteria may include members of the order Rhodobacterales and the genera *Sediminibacterium* and *Bacillus*. Rhodobacterales are primarily distributed in seawater and sediments, with increased abundance during periods of high phytoplankton proliferation, particularly in micro-eutrophic environments rich in nutrients and organic particulates. They can directly interact with algal cells and algal-derived debris particles, and they degrade many high-molecular-weight compounds such as polysaccharides and proteins (Mann et al., 2013; Barbeyron et al., 2016). Algal proliferation generally occurs during summer, and previous eukaryotic sequencing of Juvenile crab stomachs has also revealed gene sequences of certain algae such as diatoms and green algae; this is consistent with the function of Rhodobacterales in the gastrointestinal tract. Members of *Sediminibacterium* are strict aerobic bacteria commonly observed in freshwater, seawater, and sediments, and they may play a role in endocrine disruption and nitrogen removal (Hu et al., 2023). The main food source for juvenile crabs is sediment from the intertidal zone, which is habitat of *Sediminibacterium*. Therefore, this genus may represent an exogenous microorganism of juvenile crabs. Members of the genus *Bacillus* belong to the phylum Firmicutes and possess broad metabolic capabilities for decomposition (Li et al., 2020). They are beneficial to hosts and have a positive impact on the digestion and absorption of nutrients, immune responses, and the growth of aquatic animals (Sohaib Ahmed Saqib et al., 2023).

In the summer, potential pathogenic bacteria include members of the genera *Shewanella* and *Pseudomonas*. *Shewanella*, a gram-negative, non-fermentative rod-shaped bacterium, has increasingly been reported as a newly emerging pathogen in humans (Zhang et al., 2021); however, its pathogenicity to crustaceans remains unclear. *Pseudomonas*, a genus of aerobic, non-fermentative gram-negative bacteria, is commonly found in diverse environments and can cause various infections in humans (Sagar, 2024). *Pseudomonas* isolates exhibit excellent potential for biodegradation of most organic compounds (Sheng et al., 2022). Because juvenile crabs at Chongming Island mainly feed on sediment and the characteristics exhibited by *Pseudomonas*, there may be a symbiotic relationship between juvenile crabs and *Pseudomonas*. However, it is unclear whether *Pseudomonas* is pathogenic to juvenile crabs.

During winter, potential pathogenic bacteria included Firmicutes bacteria that belong to the class Mollicutes, which are very small wall-less pleomorphic bacteria (Woese et al., 1980). They are widely distributed in various ecological niches, have symbiotic relationships with some mollusks, and are the primary contaminants of cell cultures and vaccines (Chernova et al., 2021).

The number of specialized bacterial groups was significantly higher in summer compared with autumn and winter. The different living environments and functions of various bacterial groups may indicate that they play roles in juvenile crab intestines. Because Chinese mitten crab juveniles are small and their main food source is sediment from intertidal zones, they are more susceptible to the influence of environmental bacterial communities compared with their endogenous microbial communities. These bacterial communities maintain a symbiotic relationship with juvenile crabs, and whether they are pathogenic requires verification by single-strain culture infection experiments.

### 4.3 Seasonal variations in the symbiotic network of juvenile Chinese mitten crab intestinal microbiota

Symbiotic network analysis is a method that examines potential interactions between organisms and their microbiota in complex natural communities. Topological characteristics of networks, such as the number of nodes and edges, average degree, average path length, modularity, and clustering coefficient, can provide insights into the fundamental properties of networks. A previous study found that larger values associated with topological features (such as average degree, density, and clustering coefficient) indicate more connections in the network; a greater clustering coefficient degree supports more network connections, which shows a positive correlation with the interaction strength between microorganisms (Guo et al., 2022). Furthermore, a higher ratio of edges to nodes indicates greater susceptibility of the network, whereas decreases in modularity and average path length represent greater environmental pressure and worse network stability (Niu et al., 2022). Environmental disturbances are more likely to spread to other parts of the microbial community (Hernandez et al., 2021), and the lower modularity in autumn indicated that network stability changed during this season, possibly due to factors such as temperature.

Furthermore, establishing positive feedback loops between populations can help elucidate their adaptability. Removal of nodes with high betweenness centrality from the microbial network may lead to deterioration in the stability of the microbial community. During the transition from summer to autumn, the ecological niche shifted, and some Tenericutes species replaced Proteobacteria species. Internal competition may be one of the reasons for the decreased stability during autumn.

In ecological network analysis, each module typically represents ecological niches with similar functions (Williams et al., 2014). With increasing stress, competitive groups involved in interspecies antagonistic interactions are replaced by stress-tolerant species. In summary, changes in ecological niches occurred during the transition from summer to autumn, and the reasons for these changes need to be further explored in future studies (Garcia et al., 2020; Yang et al., 2024).

## 5 Summary

This study included a compositional analysis of the seasonal dynamics of juvenile Chinese mitten crab intestinal microbiota. The differences between the upstream and downstream sites on Chongming Island were not significant, which indicated that there might not be significant changes among similarly sized crab populations during their upstream migration in the Yangtze River Estuary. Prior studies showed that intestinal microbiota of river crab populations in different regions of China may exhibit differences due to environmental variations. In this study, the intestinal microbiota of juvenile crabs in different areas changed over the seasons. Seasonal diversity analysis of all crabs revealed specialized compositions of intestinal microbiota in spring, summer, and autumn, which allowed them to adapt to the current environment. Additionally, previous sampling and dietary analyses showed that the Chongming Island environment varies over seasons; this affects the food sources for juvenile crabs. Temperature and other factors contribute to these environmental differences, which prompts adaptations of the specialized microbial communities and network structures within the crab intestines to cope with seasonal changes.

## Data availability statement

The datasets presented in this study can be found in online repositories. The names of the repository/repositories and accession number(s) can be found below: https://www.ncbi.nlm.nih.gov/, PRJNA1114298.

## Ethics statement

The animal study was approved by the scientific ethics committee of the East China Sea Fisheries Research Institute. The study was conducted in accordance with the local legislation and institutional requirements.

## Author contributions

ZQ: Data curation, Formal analysis, Investigation, Visualization, Writing – original draft, Writing – review & editing. SW: Funding acquisition, Project administration, Resources, Supervision, Validation, Writing – review & editing. YW: Data curation, Investigation, Methodology, Writing – original draft. JS: Supervision, Validation, Writing – review & editing. FZ: Funding acquisition, Project administration, Resources, Supervision, Writing – review & editing.
